# Supplementary figures and images for: Re-use of laboratory utensils reduces CO2 equivalent footprint and running costs
Source: PLoS One. 2023 Apr 12;18(4):e0283697. doi: 10.1371/journal.pone.0283697 (PMC10096514; doi:10.1371/journal.pone.0283697)

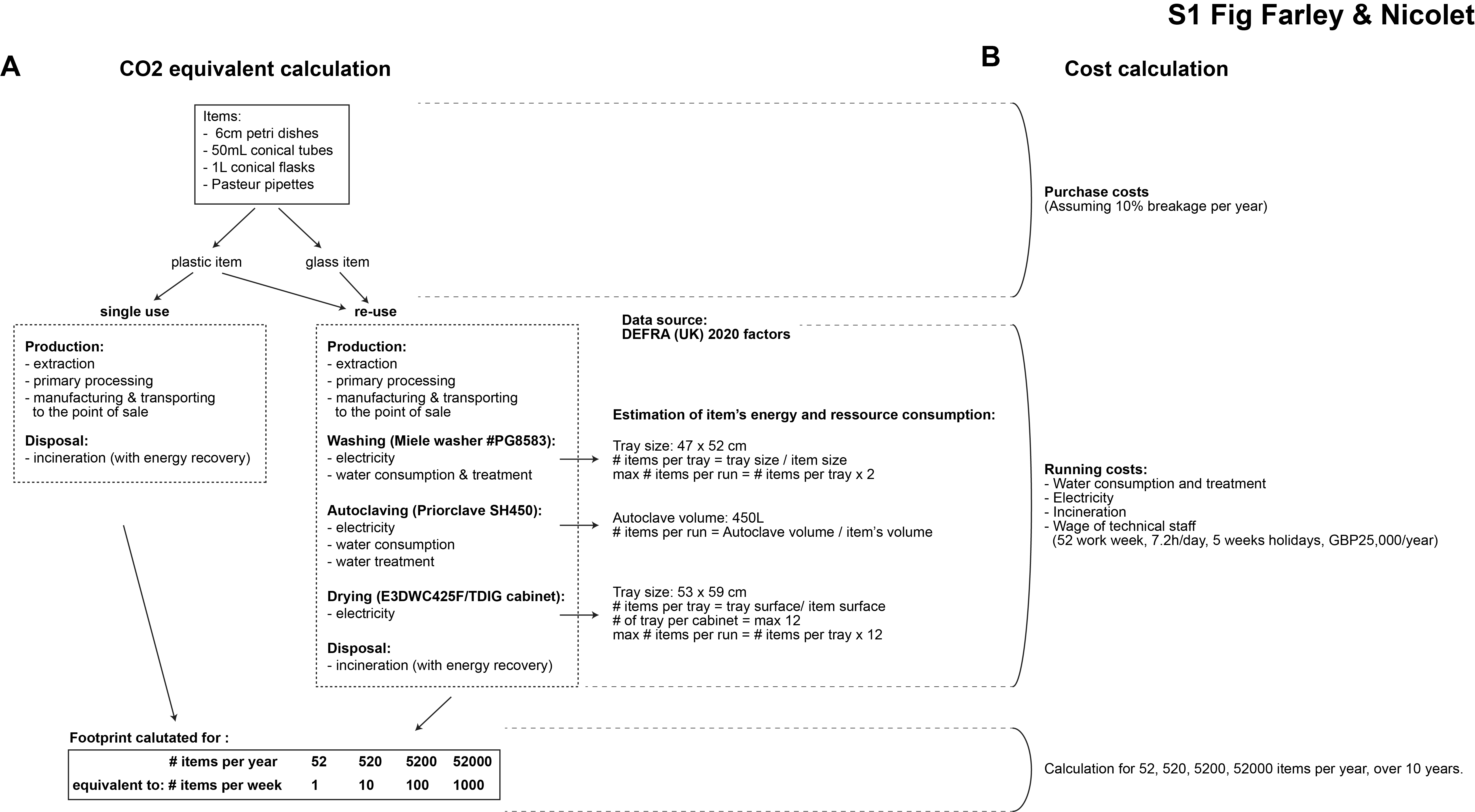

Supplement: S1 Fig — (A-B) Representation of the calculation methodology for (A) CO2 equivalent and (B) costs (see Materials and methods). (JPG) [file pone.0283697.s001.jpg]

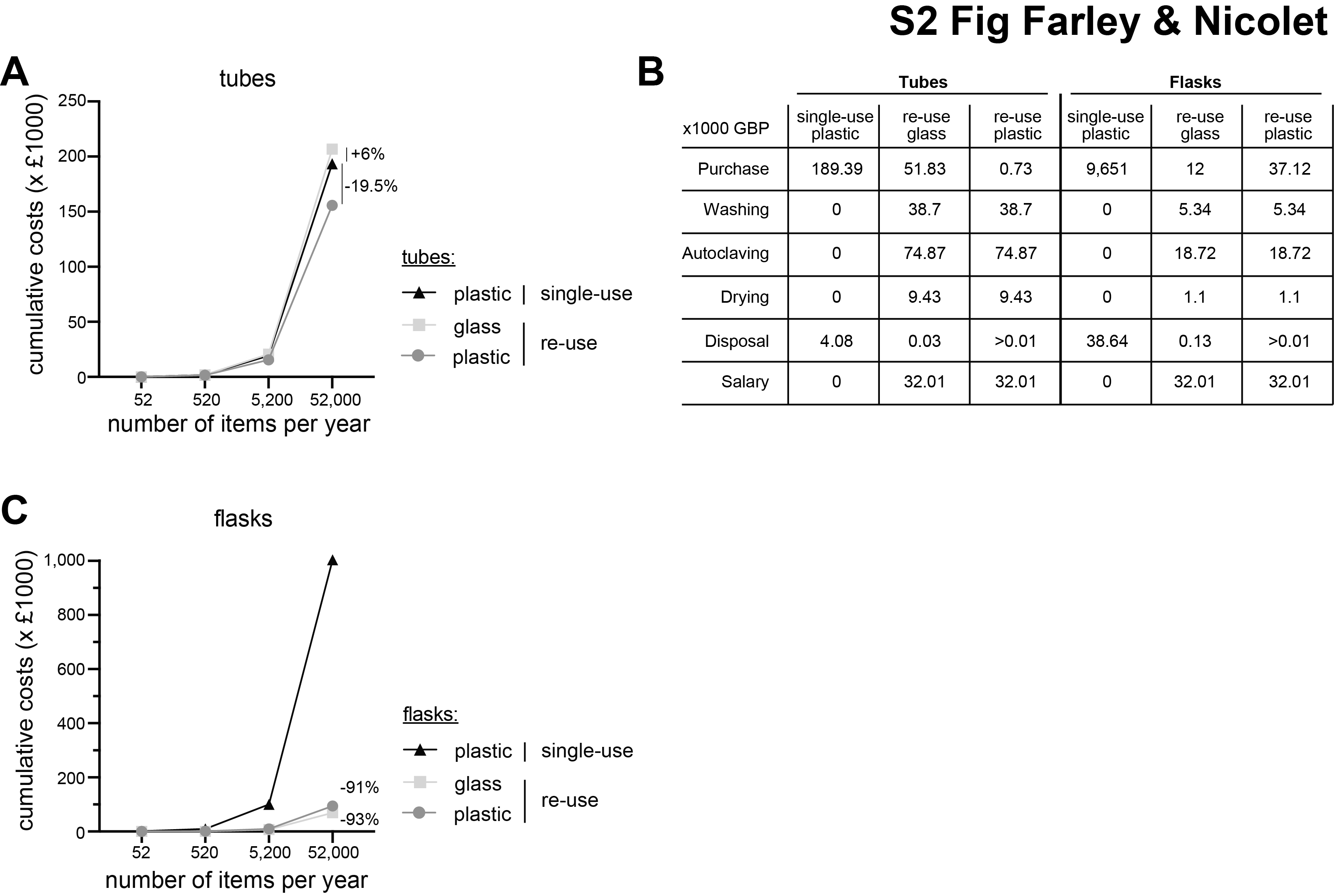

Supplement: S2 Fig — (A-C) Running costs of a 10-year period for (A-B) 50mL conical tubes and (B-C) conical 1L flasks including costs associated with salary of support staff for re-use scenario. (B) Presents the details of (A,C) in x1000 GBP for the scenario of 52 000 items used per year. (JPG) [file pone.0283697.s002.jpg]
