# Supplementary material for: Re-use of laboratory utensils reduces CO2 equivalent footprint and running costs
Source: PLoS One. 2023 Apr 12;18(4):e0283697. doi: 10.1371/journal.pone.0283697 (PMC10096514; doi:10.1371/journal.pone.0283697)
Supplement: Attachment — Submitted filename: Review Report Plos One_PONE-D-22-26188.pdf [file pone.0283697.s004.pdf]

<u>Review Report</u>

Paper Title: Re-use of labware reduces CO2 equivalent footprint and running costs in laboratories

The authors have attempted to test the effect of a concept related to circular economy, which is truly a time-demanding topic. The overall work is good. However, I feel that there is still some room to improve the paper before it can be published. My suggestions are as follows:

Abstract:

1. The abstract is well-written, clarifying the reasons of this study and highlighting the main findings.

Introduction

1. Please include page numbers and/or line numbers.
2. The background information is insufficient and needs to be elaborated. The authors should be able to build up a concrete and coherent relationship between the circular economy of labware use, its effect on CO2 emissions and other environmental benefits along with the economic gains of the reuse. And then the research gaps need to unfolded step by step. Please include one or two paragraphs detailing this information.
3. The novelty statement is missing and the claimed novelties have been posted without any detailed literature review. I suggest writing the originalities of this article point-by-point accounting the recent progress in the literature. This is section where the authors need to show how they stand out from other existing papers in the scholarship.
4. I do not see any separate literature review section. Also, the introduction section does not dive deep into the existing literature.
5. Please number the sections and sub-sections accordingly.

Methods

1. The method section looks very abstract. It would be great if the authors could include a pictorial flowchart detailing the methodological framework used in this paper.

Empirical Analysis and Conclusion

The results and discussion sections are well-written. However, the conclusion and policy implications are missing. Please include these and specify the limitations of this study. Along with that suggest for the future studies.
